# Endovascular Treatment of a Pancreaticoduodenal Artery Aneurysm in the Context of Celiac Trunk Occlusion

**DOI:** 10.7759/cureus.99424

**Published:** 2025-12-16

**Authors:** Karel Peper, Stefan Debatin, Marius K Hartmann

**Affiliations:** 1 Interventional Radiology, Klinikum Darmstadt, Darmstadt, DEU

**Keywords:** celiac trunk occlusion, coil embolization, endovascular treatment, flow diverter, pancreaticoduodenal artery aneurysm

## Abstract

Pancreaticoduodenal artery aneurysms (PDAAs) are rare but potentially life-threatening due to the risk of rupture. Their management is particularly challenging in cases of tortuosity of the parent artery and concurrent celiac trunk occlusion, where collateral circulation must be preserved. We report the case of an 81-year-old female with an 18-mm aneurysm in the pancreaticoduodenal artery, discovered incidentally during a routine liver ultrasound in hepatitis C follow-up. The patient was highly concerned about the risk of rupture but declined open surgical repair. Imaging confirmed celiac trunk occlusion with collateral perfusion via the superior mesenteric artery (SMA). Due to the vascular anatomy, direct catheterization was challenging. A steerable sheath was used to access the parent artery, allowing the deployment of a flow diverter stent to preserve blood flow in the parent artery while coiling the aneurysm in jailing technique. Post-procedural imaging confirmed successful aneurysm occlusion with preserved collateral circulation. The patient was discharged in good condition after 48 hours. This case highlights the technical considerations and alternative endovascular strategies required for managing PDAAs in the presence of tortuosity of the parent artery and celiac trunk occlusion. A combination of flow diversion and coil embolization was effective in aneurysm occlusion while maintaining collateral circulation and preventing rupture.

## Introduction

Pancreaticoduodenal artery aneurysms (PDAAs) are rare, accounting for less than 2% of all visceral artery aneurysms. The majority of these aneurysms are associated with underlying vascular anomalies, including stenosis or chronic occlusion of the celiac trunk, which leads to increased collateral flow through the pancreaticoduodenal arcade, predisposing it to the formation of aneurysms [1,2].

PDAAs are often discovered incidentally, but their rupture carries a high mortality rate of up to 50% [3,4].

The management of these aneurysms is particularly complex in patients with celiac trunk occlusion, as the pancreaticoduodenal artery plays a critical role in collateral perfusion of the liver, spleen, and stomach. Endovascular repair is increasingly preferred over open surgery due to its minimally invasive nature and ability to preserve collateral pathways [5].

This case highlights the technical challenges encountered in treating a pancreaticoduodenal artery aneurysm in an 81-year-old female with celiac trunk occlusion, ultimately requiring a combination of flow diversion and coiling for successful exclusion.

## Case presentation

Patient history and presentation

**Figure 1:**
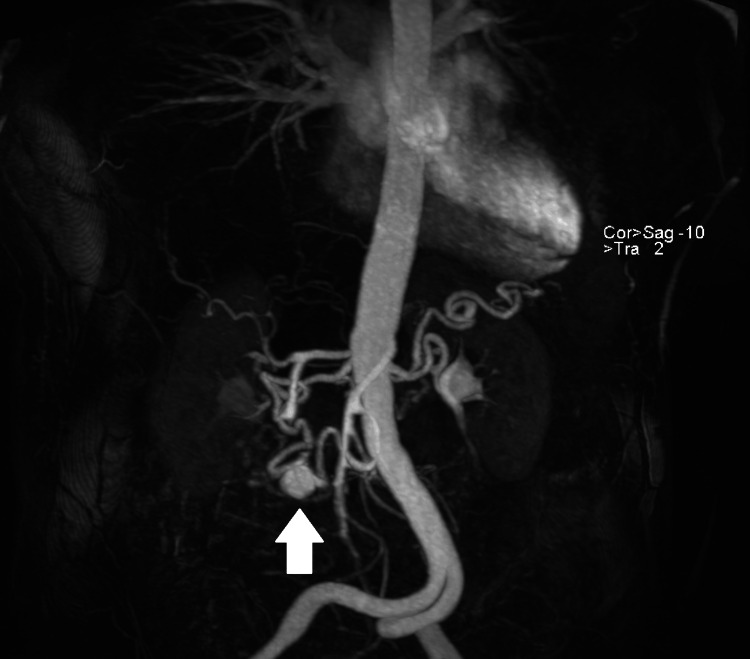
Maximum-intensity-projection (MIP) from a contrast-enhanced MRA demonstrating a visceral aneurysm of the pancreaticoduodenal artery (arrow) with retrograde opacification of the hepatic artery, splenic artery, and other celiac trunk branches via collateral flow from the superior mesenteric artery (SMA).

An 81-year-old female with a history of hepatitis C underwent routine abdominal ultrasound, which revealed an incidental pancreaticoduodenal artery aneurysm. MRI confirmed an 18-mm aneurysm, located 157 mm from the superior mesenteric artery (SMA) origin, in the context of celiac trunk occlusion (Figure 1).

**Figure 2:**
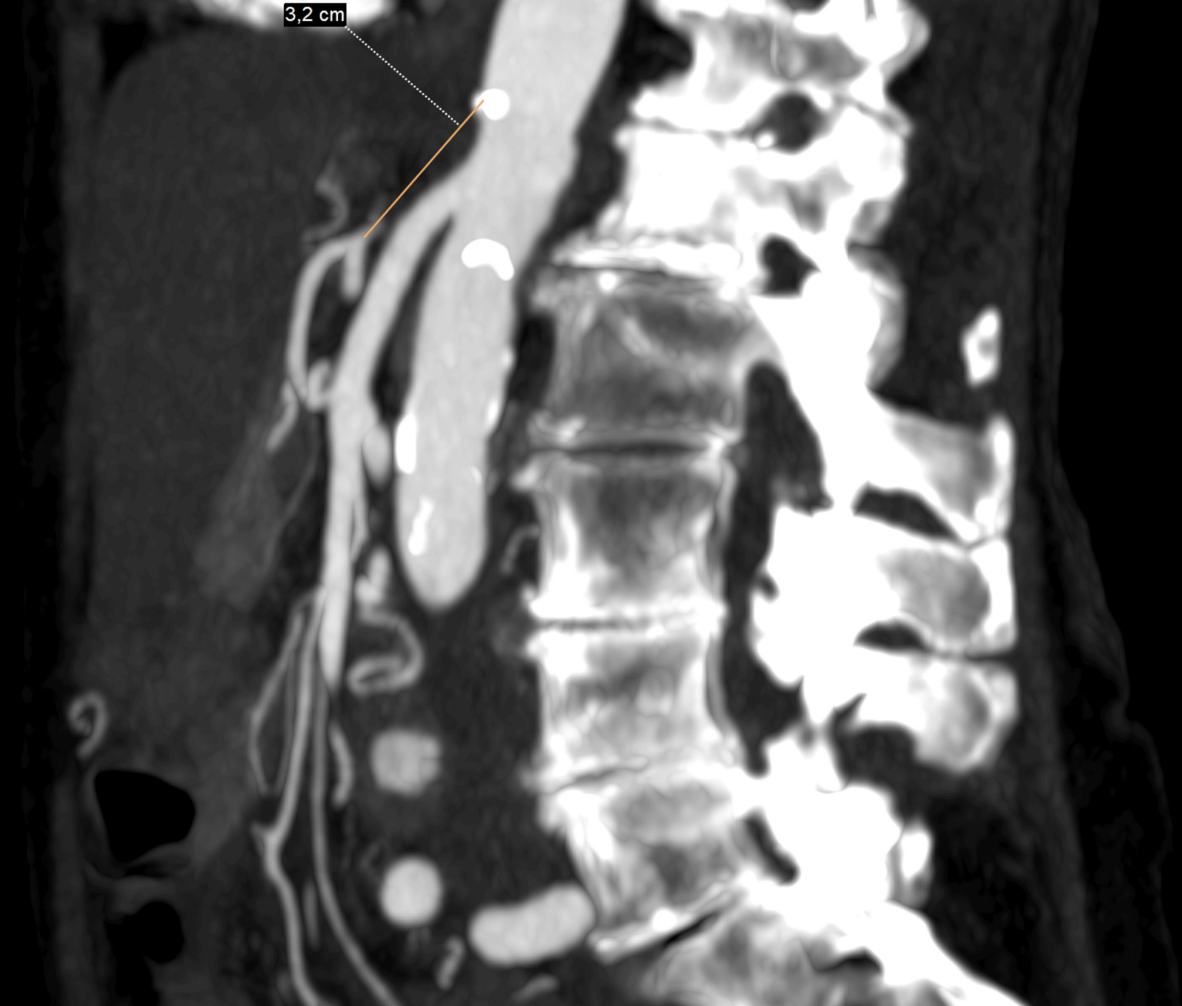
CT angiography sagittal maximum-intensity-projection (MIP) demonstrating a steep aortomesenteric angle and complete long-segment chronic occlusion of the celiac trunk without a residual ostium.

An additional CT angiography demonstrated complete occlusion of the celiac trunk over a length of approximately 32 mm, without any discernible aortic ostium or residual stump. This represents a long‑segment chronic occlusion rather than a short focal stenosis. As a result, the hepatic, splenic, and left gastric arteries were perfused retrogradely through the pancreaticoduodenal arcade supplied by the SMA (Figure 2).

Because no low-risk re‑entry point was identifiable and endovascular recanalization was technically unfeasible, celiac revascularization or stenting was not considered. The patient was concerned about rupture but is strongly opposed open surgical repair.

Endovascular procedure

Access and Challenges

The patient was preloaded with dual antiplatelet therapy (acetylsalicylic acid 100 mg and clopidogrel 75 mg) approximately one hour before the flow-diverter placement to prevent acute thrombosis.

Right common femoral artery (CFA) access was obtained using a 7Fr sheath (Destination, 7Fr, 45cm, Terumo) as a covered stenting with a self-expandable stentgraft (Viabahn; Gore, Sanat Clara, CA, USA), was originally planned.

**Figure 3:**
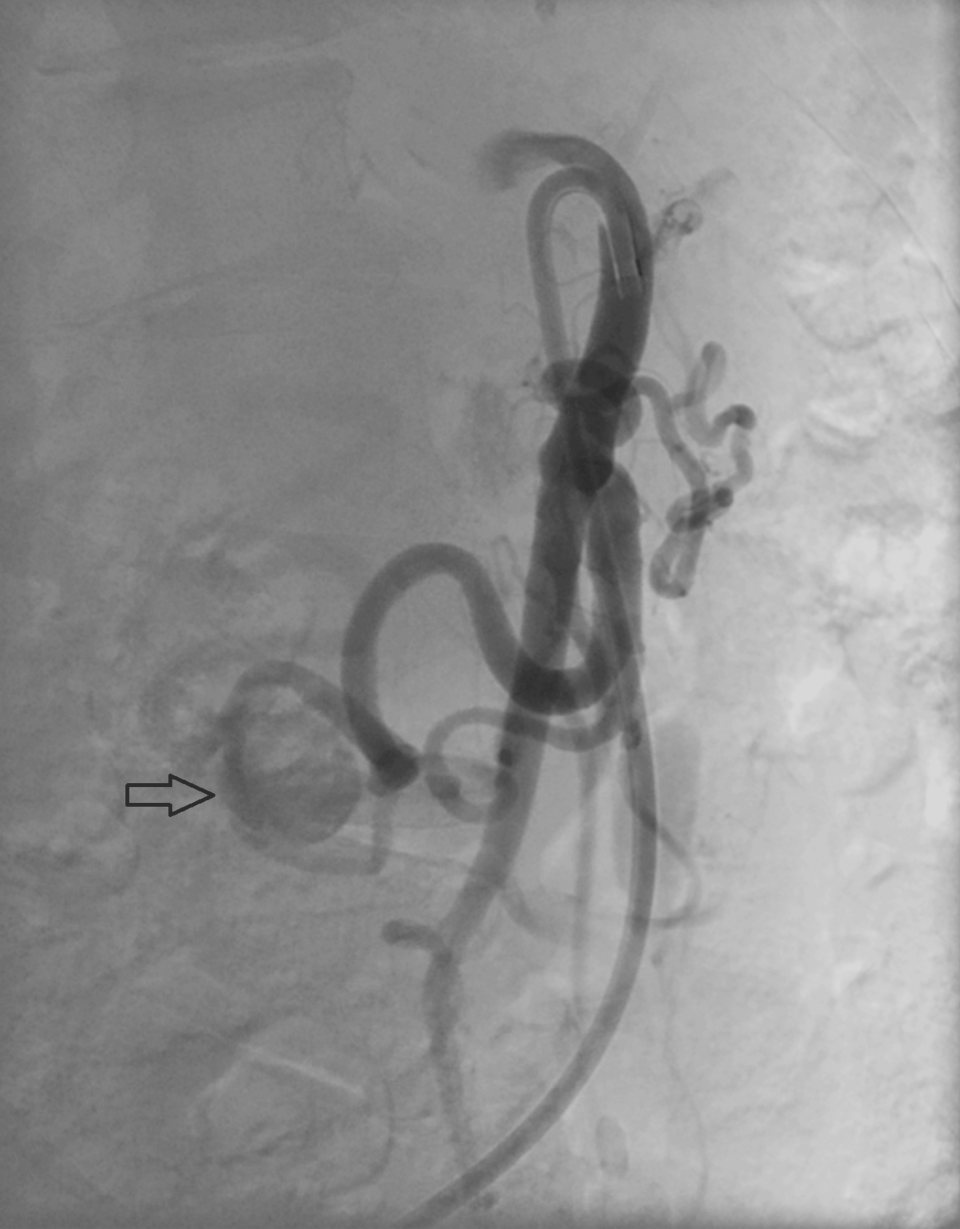
Digital subtraction angiography via a steerable sheath in the proximal superior mesenteric artery (SMA) demonstrating an elongated pancreaticoduodenal artery (arrow) with aneurysmal dilatation, and retrograde opacification of the gastroduodenal artery and common hepatic artery.

Due to the acute aortomesenteric angle seen from the femoral retrograde approach, standard catheterization techniques failed. A steerable sheath (Destino Twist, 6.5Fr, 65cm, Oscor, Palm Harbor, FL, USA) was required to navigate the SMA origin (Figure 3); however, this sheath was incompatible with the planned covered stent graft (Viabahn) because of its smaller inner diameter (6.5Fr versus 7Fr required).

Because the hepatic, splenic, and gastric arteries were entirely dependent on retrograde flow through the pancreaticoduodenal arcade, sole coiling was avoided to minimize the risk of coil migration and unintended occlusion of this vital collateral pathway.

Six tortuous bends from the SMA to the aneurysm and an acutely angulated SMA origin (163°) were additional considerations prompting the choice of a different approach with a flow diverter stent and coiling using the jailing technique, as the planned Viabahn or other vascular stents and stentgrafts are relatively rigid and would have been difficult to navigate and accurately position across the aneurysm ostium. Flow diverters can be delivered through a microcatheter system, which allows for easier navigation in the markedly tortuous and narrow pancreaticoduodenal arcade.

**Figure 4:**
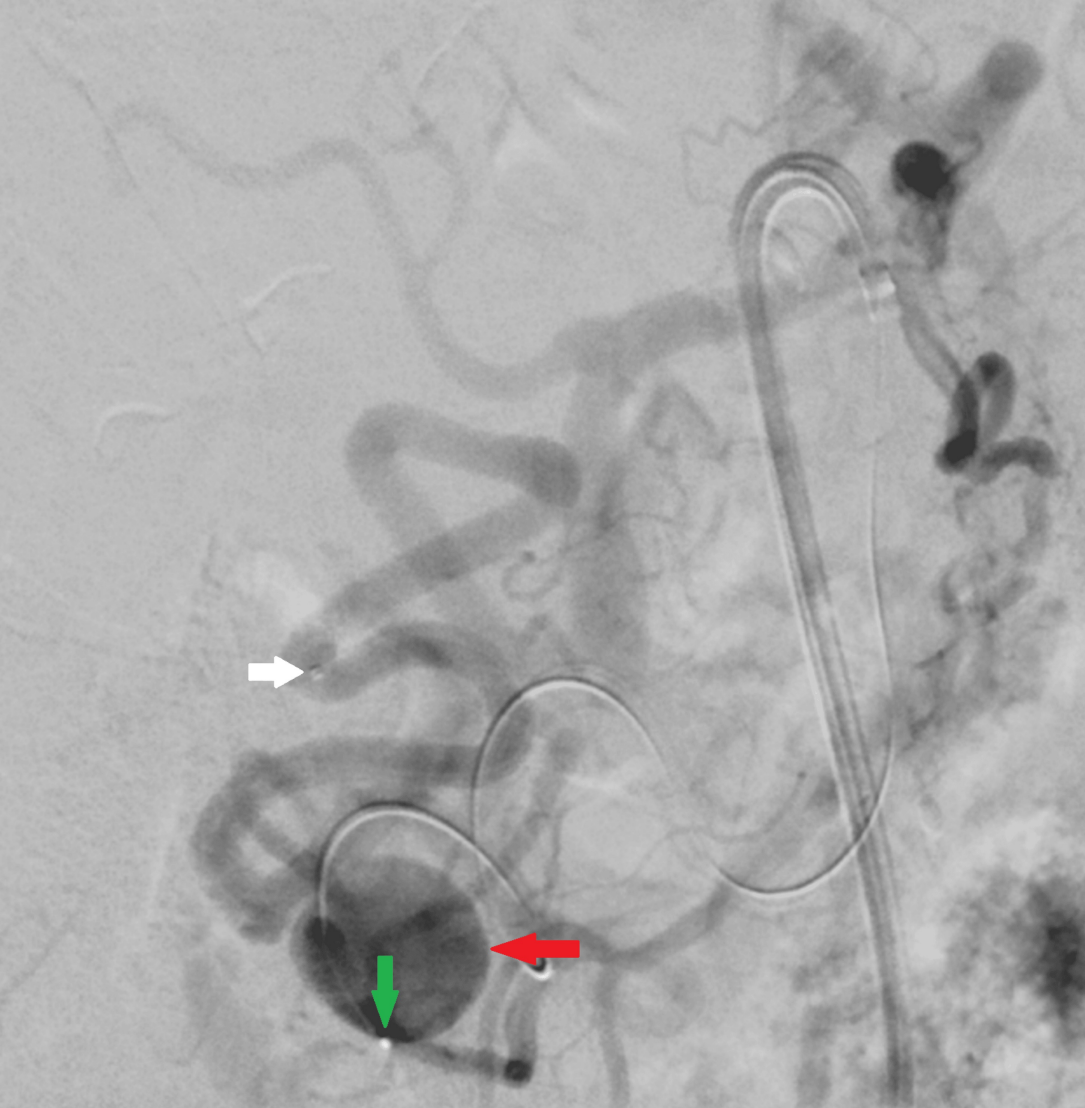
Digital subtraction angiography via a steerable sheath in the proximal superior mesenteric artery. A first microcatheter is advanced distal to the pancreaticoduodenal artery aneurysm into the gastroduodenal artery (white arrow), while a second microcatheter (green arrow) with a 0.014-inch microwire is positioned within the aneurysmal sac (red arrow) of the pancreaticoduodenal artery .

First a microcatheter (Phenom 27, 150 cm, Medtronic, Minneapolis, MN, USA) was navigated over a 0.014” microwire (Transend 205 cm, Boston Scientific, Marlborough, MA, USA) distally to the aneurysm in the pancreaticoduodenal artery (Figure 4).

Thereafter a second microcatheter (Renegade STC, 150 cm, Boston Scientific) was placed within the aneurysm sac over a 0.014” microwire (Transend 205 cm, Boston Scientific).

The size of the flow diverter was selected according to the parent vessel diameter (approximately 4.6 mm), with an intended 20% oversizing, resulting in a device diameter of 5.5 mm. The microwire was then withdrawn from the distally placed microcatheter, and the flow diverter stent (Pipeline Vantage 5.5 × 30 mm, Medtronic) was loaded into this microcatheter.

**Figure 5:**
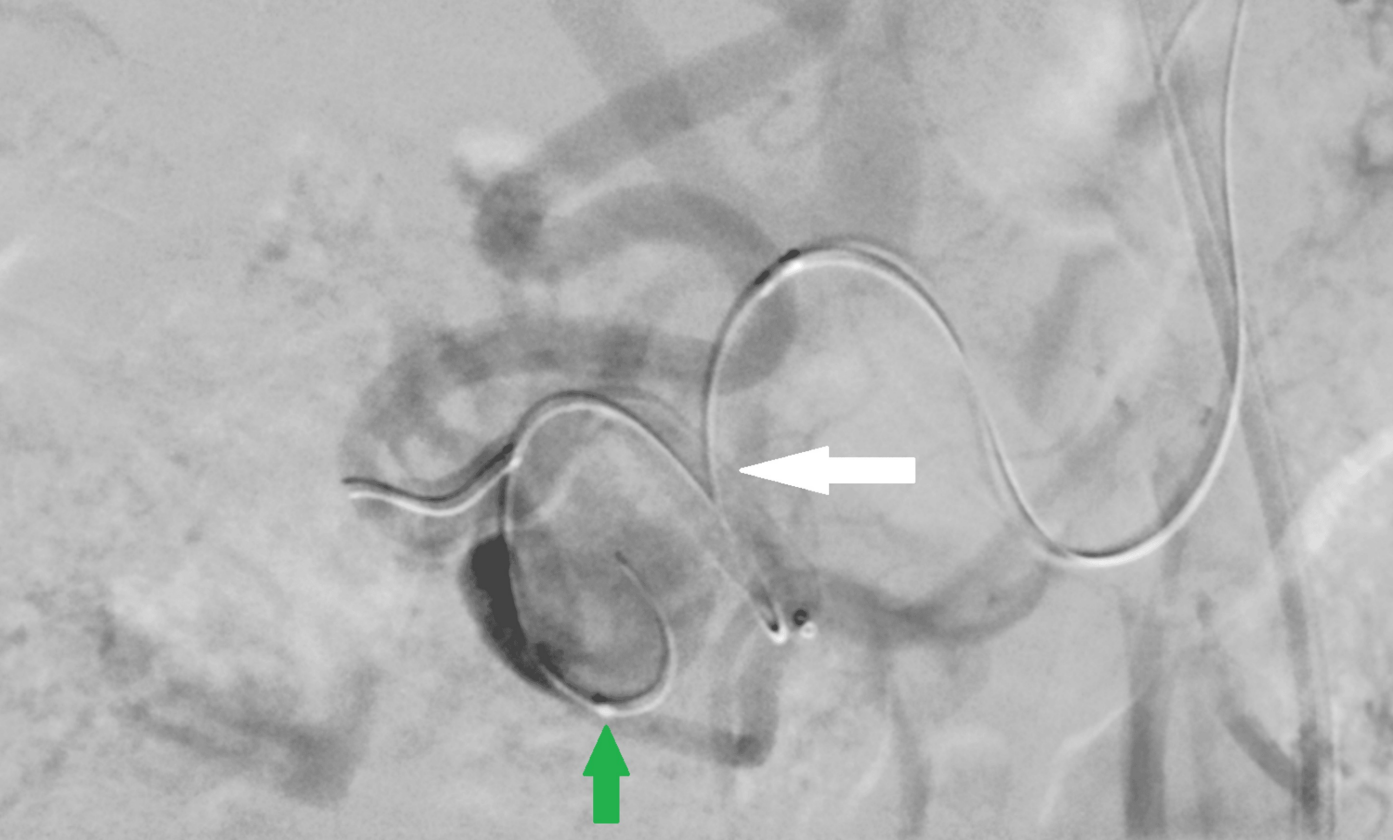
Digital subtraction angiography after deployment of a flow diverter (white arrow) across the pancreaticoduodenal artery aneurysm demonstrates markedly reduced aneurysmal filling, with preserved patency of both the gastroduodenal artery and pancreaticoduodenal artery. A second microcatheter (green arrow) and 0.014″ microwire are jailed behind the flow diverter mesh within the aneurysm sac, in position for coil embolization using the jailing technique.

Under high‑resolution fluoroscopic guidance, the device was deployed across the aneurysm neck (Figure 5). The deployment was performed using the standard push‑and‑pull technique: the distal end of the flow diverter was first released and allowed to expand against the vessel wall, after which the microcatheter was gradually withdrawn under continuous fluoroscopic monitoring to maintain accurate positioning and optimal wall apposition.

**Figure 6:**
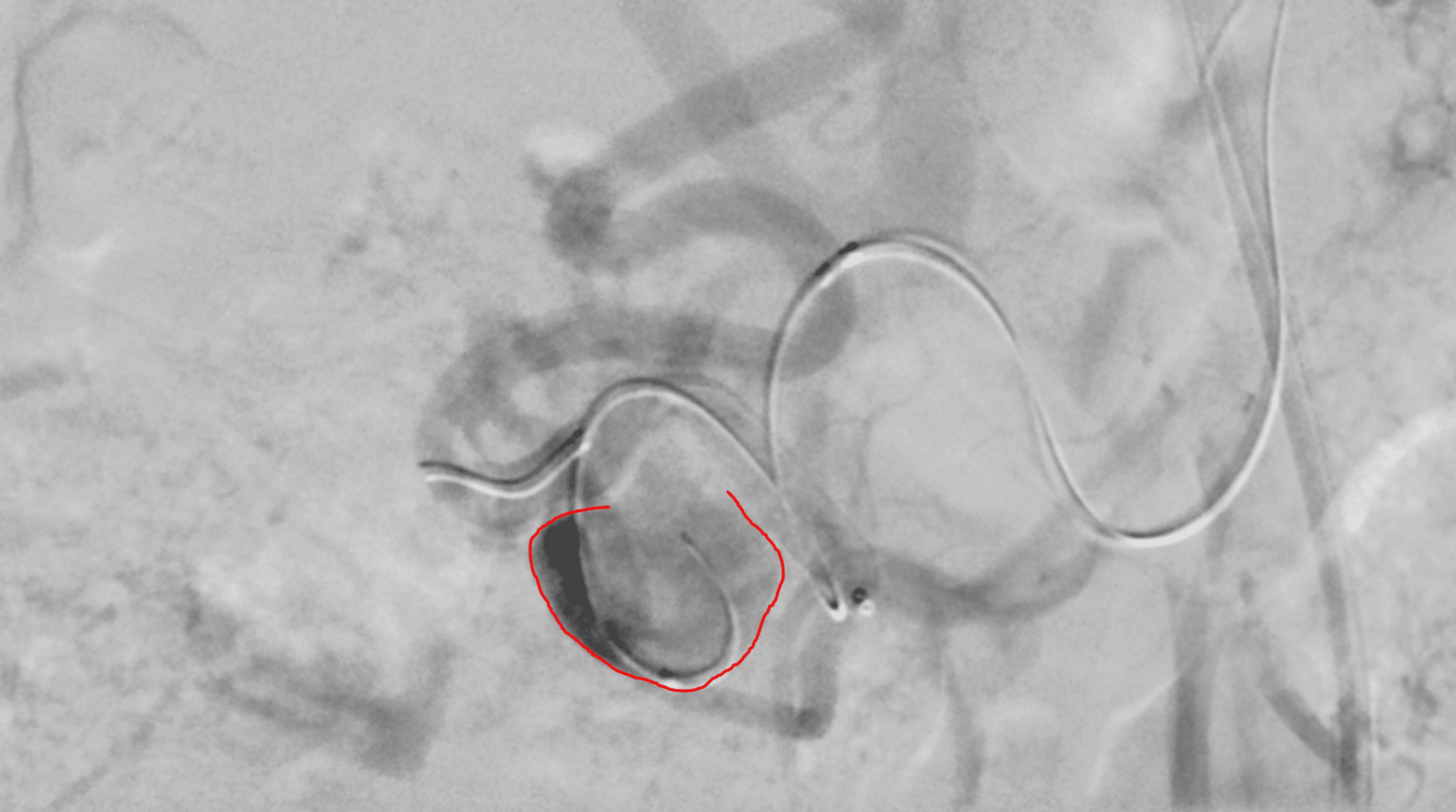
Digital subtraction angiography after flow‑diverter placement demonstrating marked contrast stagnation within the aneurysm sac (red circle), indicative of reduced intra‑aneurysmal flow and pressure. The second, proximally positioned microcatheter and microwire are also visible; they lie outside the lumen of the flow diverter with the microcatheter tip remaining inside the aneurysm, prepared for subsequent coil embolization using the jailing technique.

Although the aneurysm already showed contrast stagnation within the sac after flow‑diverter deployment (Figure 6), suggesting that a flow diverter alone might have been sufficient, a combined approach was chosen for safety and durability in this elderly patient who preferred a single definitive procedure.

**Figure 7:**
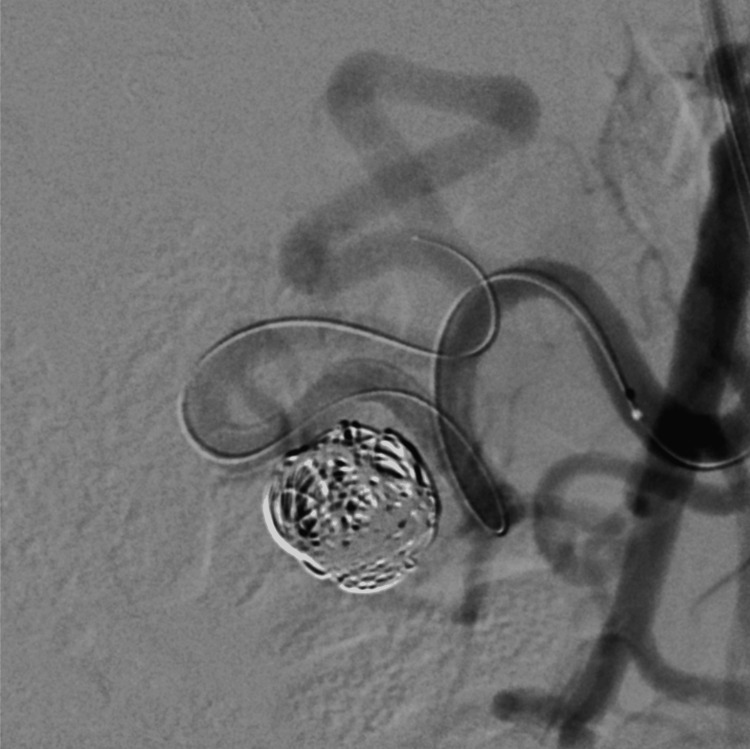
Magnified digital subtraction angiography image showing complete aneurysm occlusion, preserved flow‑diverter configuration, and maintained patency of the pancreaticoduodenal arcade. A microwire remains positioned through the lumen of the flow diverter, while the microcatheter is kept proximal to the device as a precaution before final removal.

Coil embolization was carried out using three 14 × 300 mm, two 12 × 200 mm, three 10 × 200 mm, and two 8 × 20 mm detachable coils (IDC, Boston Scientific) (Figure 7).

After coil embolization using the jailing technique, the microcatheter was carefully retracted under high‑resolution fluoroscopic guidance to ensure that the flow diverter remained in place. The detailed DSA view demonstrates successful aneurysm occlusion, preserved flow‑diverter morphology, and patency of the pancreaticoduodenal arcade (Figure 7).

**Figure 8:**
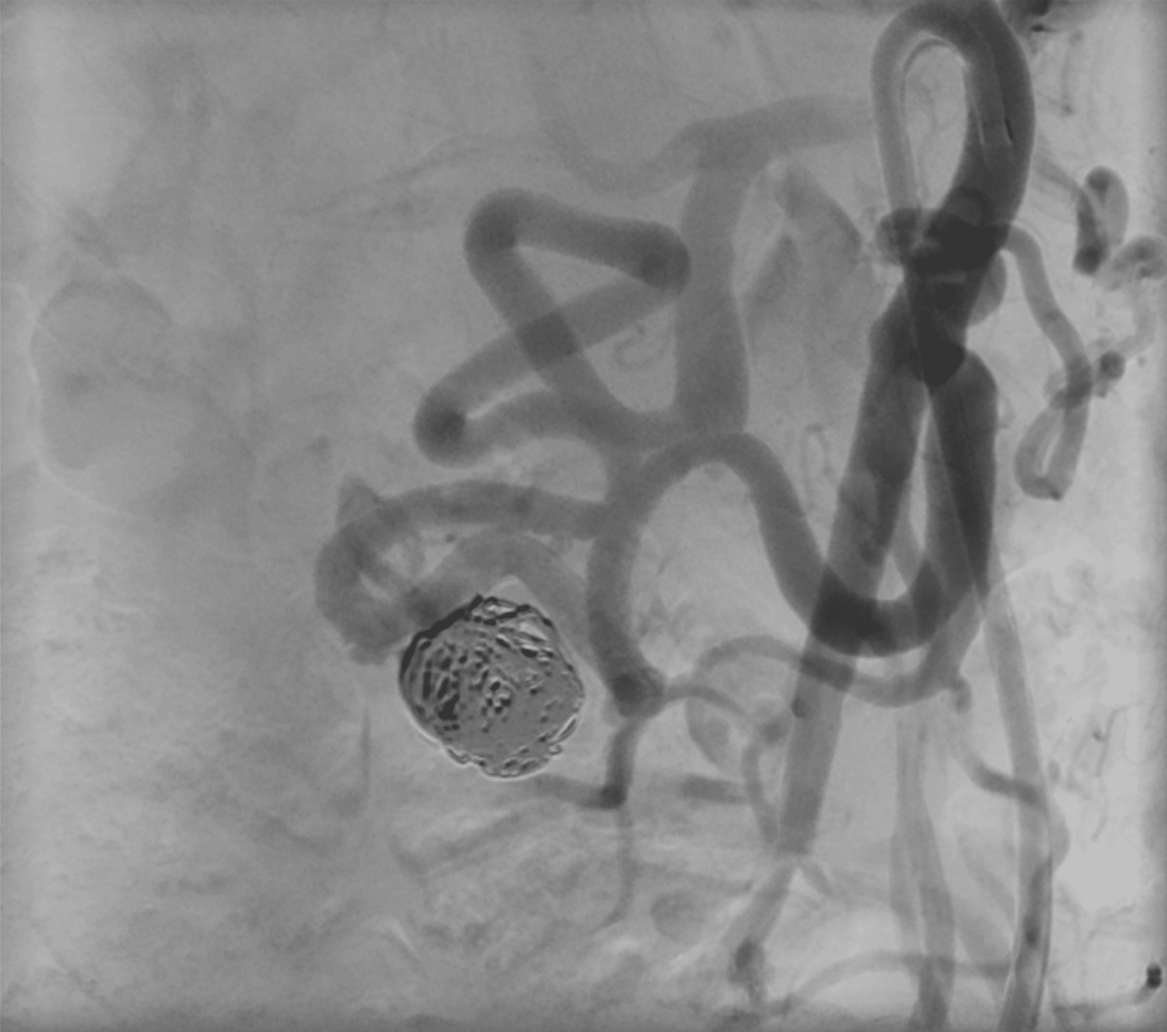
Final digital subtraction angiography demonstrating the flow diverter in situ with successful coil embolization of the aneurysm. Patency of the efferent vessels is preserved.

Final angiography confirmed complete aneurysm occlusion with preserved collateral flow (Figure 8). Hemostasis was achieved using a Closure device (AngioSeal, 8Fr, Terumo, Tokyo, Japan) for the right femoral access site. The procedure was uneventful, and the patient was discharged home within 48 hours in good condition. Dual antiplatelet therapy with acetylsalicylic acid 100 mg and clopidogrel 75 mg daily was prescribed for six months.

## Discussion

PDAAs are strongly associated with rupture, irrespective of aneurysm size or whether the aneurysm is true or false in nature. Therefore, repair is generally recommended in patients with acceptable operative or interventional risk profiles [6].

Management of PDAAs is particularly challenging in the setting of celiac trunk occlusion, as these aneurysms develop under high-flow conditions due to the recruitment of extensive collateral circulation. While open surgical repair is effective, it carries a high risk of morbidity, especially in elderly patients [7,8].

Endovascular coil embolization has recently become the preferred treatment modality, regardless of whether the aneurysm has ruptured or remains intact [9]. Covered stent placement may be considered when the proximal and distal segments of the involved artery are of appropriate diameter and exhibit minimal tortuosity [7].

Endovascular techniques offer a valuable alternative to open repair, but require meticulous planning to preserve collateral perfusion. Among these, flow diversion has emerged as a promising strategy.

Flow-diverting stents (FDS), originally developed for the treatment of intracranial aneurysms, have significantly expanded the scope of endovascular therapy. The low porosity and high mesh density of FDS promote aneurysm thrombosis by reducing intra-aneurysmal blood flow and serving as a scaffold for endothelialisation across the aneurysm neck. This leads to progressive aneurysm occlusion and lower rates of recanalization. Importantly, FDS are designed to preserve perfusion through collateral branches [10,11].

Adjunctive coil embolization can further enhance aneurysm thrombosis and minimize the risk of delayed rupture.

This case demonstrates the importance of a dual-microcatheter approach, allowing safe aneurysm exclusion while preserving critical collateral circulation.

## Conclusions

Endovascular treatment of PDAAs in the setting of celiac trunk occlusion requires a carefully tailored approach. This case demonstrates that combining flow diversion with selective coil embolization can successfully exclude the aneurysm while preserving vital collateral circulation. PDAAs pose a high rupture risk regardless of their size, emphasizing the importance of timely and strategic management. The use of flow diverters offers a means to achieve aneurysm exclusion without sacrificing essential collaterals, making them particularly advantageous in distal, tortuous, or difficult-to-access vessels. Moreover, the integration of flow diversion with adjunctive coiling through a dual microcatheter technique enhances the likelihood of complete aneurysm occlusion and decreases the risk of delayed rupture.

Together, these insights underscore that a meticulous, multimodal endovascular strategy can provide both safety and efficacy in the complex scenario of PDAAs associated with celiac trunk occlusion.
